# Making the case for investment in public health: experiences of Directors of Public Health in English local government

**DOI:** 10.1093/pubmed/fdv035

**Published:** 2015-03-15

**Authors:** M. Willmott, J. Womack, W. Hollingworth, R. Campbell

**Affiliations:** 1 School of Social and Community Medicine, University of Bristol, Bristol BS8 2PS, UK; 2 Wiltshire Council, County Hall, Bythesea Road, Trowbridge, Wiltshire BA14 8JN, UK

**Keywords:** public health, management and policy, young people

## Abstract

**Background:**

Amid local government budget cuts, there is concern that the ring-fenced public health grant is being appropriated, and Directors of Public Health (DsPH) find it difficult to make the case for investment in public health activity. This paper describes what DsPH are making the case for, the components of their case and how they present the case for public health.

**Methods:**

Thirteen semi-structured telephone interviews and a group discussion were carried out with DsPH (November 2013 to May 2014) in the Southern region of England.

**Results:**

DsPH make the case for control of the public health grant and investing in action on wider determinants of health. The cases they present incorporate arguments about need, solutions and their effectiveness, health outcomes, cost and economic impact but also normative, political arguments. Many types of evidence were used to substantiate the cases; evidence was carefully framed to be accessible and persuasive.

**Conclusions:**

DsPH are responding to a new environment; economic arguments and evidence of impact are key components of the case for public health, although multiple factors influence local government (LG) decisions around health improvement. Further evidence of economic impact would be helpful in making the case for public health in LG.

## Background

Following the passage of the Health and Social Care Act 2012, local responsibility for improving public health and reducing health inequalities in England moved from National Health Service (NHS) to local government (LG) organizations (local authorities—LAs) on 1 April 2013. There is ongoing debate about the merits of this move. It may facilitate work on socioeconomic and environmental determinants of health,^1,2^ but there are concerns about capacity and technical support for Directors of Public Health (DsPH) and that public health funds may be diverted to other LG activity due to severe budget cuts imposed by national government.^3^

In addition, there are enduring challenges for securing investment in public health. These include: a dearth of evidence on the cost-effectiveness of many public health interventions,^4^ greater expectations for cost-effectiveness of public health interventions, belief that in the long-run prevention may cost more than treatment; lengthy timeframes required for some public health interventions; a preference to relieve the suffering of ‘identifiable victims’ where resources are scarce; interest group influence; and ‘the reality that evidence alone does not drive policy’.^5^

There is extensive literature on decision-making and evidence use in public policy,^6–8^ including in public health^9–12^ which suggests that many factors influence public health decision-making, and evidence is used differently in different contexts.^10^ But as yet little academic research explores how public health decisions are made in LG.

Based on interviews and discussions with DsPH, this paper describes how DsPH make the case for investment in public health in LG. It explains what DsPH make the case for, the components of their case and how they present the case, including how they use evidence.

## Methods

All current DsPH (*n* = 16) in three centres in the Public Health England Southern region were invited by email to participate in the study, just over 10% of the 132 DsPH in England. Fourteen DsPH agreed to be interviewed; one withdrew due to work commitments, and another participant was a senior public health team member as the DPH left before the interview was carried out. Semi-structured telephone interviews were carried out with these 13 DsPH (November 2013 to May 2014), 3 were from county and 10 from unitary LAs.^13^ About half of the interviewed DsPH work in LAs with a Conservative majority, others were in LAs with no overall control, Labour or Liberal Democrat majorities.^14^ Six of the interviewed DsPH cover predominantly urban LAs, the remainder being rural;^15,16^ the size of the LAs ranges from fewer than 200 000 to over 1 million population.^17^ A minority of DsPH had been in post <2 years when interviewed; some DsPH were in interim appointments (number not provided to preserve anonymity).

The interview guide used public health activity with children and young people as a ‘tracer’ to explore DsPH's perceptions and experiences of the opportunities and challenges of the LG decision-making environment and the knowledge, skills and resources they need to make the case for investment in public health. Interviewees were asked what support would be useful in making the case for public health and developing work on multiple risk behaviours among children and young people. The interview guide was not piloted but was informed by a discussion at a meeting of DsPH in South West England, arranged by PHE (September 2013). For example, as a result of this meeting, interviewees were asked about their use of evidence.

Interviews lasted between 30 and 50 min, and were audio-recorded and transcribed verbatim. Verbal consent to participate and audio-record interviews was obtained and recorded before interviews began. All transcripts were anonymized then analysed in NVivo 10 using a combination of deductive and inductive approaches. Topics covered in the interview guide provided an initial sorting framework. Data were then analysed using elements of constant comparative method;^18^ data were coded line by line to generate descriptive and explanatory categories, and deviant cases were used to test these. Categories were also informed by literature (e.g. on evidence-based policy) and theory (e.g. resource mobilization).

Dual coding was not undertaken, but analysis was regularly discussed within the research team. Preliminary analysis of 10 interviews was presented to seven DsPH (six of whom had been interviewed) at another DPH meeting (March 2014). DsPH were invited to discuss, correct and add to the interpretation to improve its validity. Written consent was obtained from all participants before the discussion, which was audio-recorded and transcribed verbatim. The anonymized transcript was used to hone the coding and categories. The interview guide was amended for the remaining three interviews to test the refined analysis. The last three interview transcripts were analysed alongside existing data to test and refine the coding and categories and ensure saturation.

The School for Policy Studies (University of Bristol) Research Ethics Committee gave ethical approval for this project (reference 2113).

## Results

### What do DsPH make the case for?

Even over the short time covered by this research, what DsPH were making the case for shifted. For example, one interviewee described how they were initially concerned to: ‘try and get the council to understand what its new public health function was’ (int.7). However, there were two consistent issues that DsPH made the case for in terms of resource allocation, control of the public health grant and action on wider determinants of health.

DsPH reported having different degrees of control over the public health grant. One interviewee described how they had negotiated financial autonomy by ‘absorbing some local authority spend’ so that ‘within reason I have freedom of action and control over my budget’ (int. 6). In contrast, another DPH felt that LG officials presumed public health grant money would be used for services that could no longer be funded through the LG budget.

DsPH described making the case for protecting and maintaining control over the public health grant and justifying how public health resources were allocated amid external scrutiny and despite the ring-fence around the public health grant. For example, one DPH said; ‘we are having to defend our budgets and justify our budgets, whether that's with people with NHS England, or it could be with Council or it could be with the CCG* - all are potential poachers of our pie*.*’ (Discussion group participant) [*Clinical Commissioning Group]. Another DPH described how in the context of overall reductions in LG spending there was pressure to explain: ‘why we're investing at this level and what outcomes we're getting’ (int. 7).

DsPH described making the case for activity at different levels that influence health,^19^ for example making the case for services providing lifestyle interventions as well as influencing activity on community safety and school transport. Some DsPH perceived that pre-conceptions about public health created an environment that was more conducive to making the case for some public health approaches and population groups than others. As one interviewee said:

*I would say politically there are certain things that they're quite happy to look at like* [*…*] preventing children from starting to smoke. That seems to be well accepted and supported. But when we look at say, provision of children's centres or early years … we are being challenged on, well, why are we investing? (int.7)

In contrast, some DsPH felt that LG was open to different models of public health. For example, one DPH described how members of the public health team were working part time in another LG department on the wider determinants of health.

DsPH reported making the case for health to be considered in wider LG budget decisions. For example, arguing for health impact assessments of proposed service cuts. DsPH also described advocating for the maintenance of population-wide as well as targeted services as they felt this was endangered in the constrained financial context. As one interviewee described: *‘*like Marmot, you know, talked about the proportionate universalism and I think that we've just got to make sure that that's maintained…’ (int.9).

### The components of the case

DsPH made positive business cases outlining needs, solutions and their effectiveness, cost and economic impact. Interviewees were clear that ‘having the economic argument is hugely important’ (int.12). This included various measures of economic impact (e.g. return on investment, cost-effectiveness) but as one interviewee explained, in the current economic context: ‘it all comes back to not just cost effectiveness but cash releasing savings’ (int.9). One interviewee gave the example of the economic case required:

*we're showing that the family-led partnerships are generating savings of more than five times the programme costs. … statements like that are of interest and they can be good for making the case and lobbying, but people* [Councillors] *want to be really, really clear who gets the savings and over what timeframe, how secure, how certain are they*. (int.13)

Evidence was perceived to be vital in substantiating arguments. As one interviewee said, evidence is ‘one of the tenets of our profession’ (int. 8). There was ‘a whole sort of list of things’ (int.5) interviewees defined as evidence, from: ‘peer reviewed and critically appraised’ evidence (int.9) to ‘examples of good practice and where we know other councils have done the work…’ (int.7). DsPH felt public health evidence is patchy, especially in demonstrating shorter term impact, savings, effects on wider determinants of health and benefits for LG. They also expressed concern about the lack of capacity to sift through the volume of evidence. To this end, interviewees mentioned several examples of sources of collated and synthesized evidence which they found useful.^20,21,22^

The evidence DsPH used differed depending on what they were making the case for and to whom. For example, a ‘service modification’ required less rigorous evidence than a new commission when allocating the public health budget (int.8). Interviewees perceived that evidence has a different status in LG than in the public health field. As one interviewee explained, they felt that their analysis of evidence was done, ‘in a public health way’ which they felt was ‘far more thorough than with what you have within the local authority’ as they believed that in LG; ‘evidence is there to influence and support, [the] political agenda perhaps and the particular area of work’. (int.6).

Several interviewees felt that evidence was not always required to support arguments where the case was congruent with current ideas. As one said; ‘a lot of the conversations we have around better multiagency working and the value for money […] you don't need to cite the evidence because it's common sense’ (int.8). Furthermore, DsPH emphasized the importance of normative arguments. As one interviewee explained;

by and large the politician's first interest is not the evidence. Or even the return on investment. Um, their first interest sits between doing the right thing and being politically acceptable. And you have to have to meet those two targets first*…* (int.6).

DsPH described responding to this by making arguments related to the reputation and accountability of politicians and the Council. For example, one interviewee mentioned it was useful to: ‘connect into something that's affecting their, in an adverse way, their population, they usually can buy into that’ (int.4). Another said they highlight activities that ‘look good for the Council’ (int.12).

Some discussion group participants perceived they were influencing LG attitudes towards evidence. As one participant remarked: ‘they [Councillors] quite like flashing evidence now’. Likewise some felt they had been influenced by the imperative for democratic accountability. As one discussion participant said: ‘the voice of the child and young person […] now probably bears far more weight than it ever did from the NHS’ and when building a case they were: ‘doing work that we didn't do in the NHS around gaining the voice of the end users’.

### Communicating the case

DsPH described how the public health team analysed, translated and summarized complex evidence for Councillors and other stakeholders. As one interviewee said; ‘if it's from a credible source, if it's a headline then usually that's as much as is needed’. (int.10). They also highlighted the importance of making evidence locally relevant, carefully framing their message (in documents or verbally) and of being ‘versed in council terms’ (int.4) to make ‘things really accessible’ (discussion participant) but at the same time, trying to ‘not come in too expert’ (int.3) so as not to repel potential allies.

DsPH felt that ‘telling stories’ about individual people was effective in ‘catching the imagination, that this is real people on your patch’ (int.4) to ‘get down to what it means to them [local Councillors]’ (int. 9). For example, a discussion participant described using a new media video at a meeting to present a: ‘patient voice slot, you know, those sort of things can be really powerful and they're about patients and you can back it up with a few facts, you know, you can kind of very easily spread a message*’*.

Another discussion group participant summarized other DsPH's perceptions of the importance of case studies in presenting a persuasive case: ‘a lot of our Councillors will be won over by even a single case where you can articulate really, really clearly in a practical everyday way what added value you have brought to bear’.

## Discussion

### Main finding of this study

The cases DsPH make for public health and how they make them attest to the delicacy of trying to exert influence as a newcomer in an organization with a constrained economic context and an overtly political system. Findings show that DsPH are responding with political sophistication: negotiating autonomy and influence; navigating pre-conceptions about public health; and framing their expertise to foster legitimacy while building relationships.

Findings confirm that knowledge is a critical resource for public health but substantiate concerns^4^ that evidence of economic impact of public health activity on wider determinants of health, the effectiveness of complex interventions and impact over the short and medium-term is urgently required, particularly as the boundaries of public health are contested in LG.

### What is already known on this topic

There is little published empirical research on the new public health system. Findings broadly concur with investigations by professional bodies^23^ and journals^24^ that have found: DsPH are generally optimistic about the development of public health in LG, but the capacity of public health teams is limited;^23^ the economic and political context of LG determines decision-making;^25^ and control of the ring-fenced budget is negotiated and contingent.^23,24,26^ The findings are consistent with the myriad literature on evidence and policy. For example that many factors influence decisions;^27–30^ multiple types of evidence are used^31^ and evidence synthesis may be a useful way to ensure research is used.^32^

### What this study adds

This study shows that despite the importance placed on evidence-based practice, normative arguments are critical in advocating for investment in public health, and as at a national level,^33,34^ well-presented ideas are persuasive in LG. This study contributes to understanding of the political influences on local public health activity which it has been argued, is ‘not yet well understood’ in the research literature and reinforces the suggestion that public health researchers need to understand the political context in which DsPH work.^6^

It substantiates literature that shows evidence is only one determinant of public health policy^28,29^ and that evidence is often broadly defined;^31^ but it also finds that DsPH are persuading LG decision makers of the value of their expertise and of evidence-based decision-making^35^ while also adapting their practice to include forms of knowledge perceived as legitimate in LG decision-making such as individuals' lived experiences. In so doing, it begins to contribute understanding of the evolving processes of public health decision-making^27^ and the importance of narrative and framing information to be persuasive and transfer knowledge.^36^

### Limitations of this study

A limitation of this study is that it explored activity in a dynamic environment, so interviewees' situations may have changed during and since data collection. Moreover, interviews were conducted over a few months due to participant availability so interviewees were experiencing different stages of the planning and budget cycles when interviewed. However, the discussion group allowed DsPH to reflect on this, as one participant remarked: ‘I am probably a little bit more positive than I was when I was interviewed…’

A second limitation is that the small sample size inhibits study generalizability. However, findings are congruent with recent surveys (see above), and the sample includes DsPH with varied tenure and in a range of LAs.

A third limitation is that the study does not explore perspectives of other stakeholders or assess the impact of cases that DsPH made; these could be explored through further research.

## Conclusions

This study provides insight into how DsPH are responding to a new context and advocating for public health investment. While it is not the only tool DsPH use to influence LG decisions, evidence of impact is key for making the case for public health. Further evidence of the economic impact of public health activities, especially over the short term, would be helpful as DsPH feel pressure to show savings to enable reinvestment and demonstrate how public health contributes not only to health, but to LG's ‘bottom line’. As one DPH remarked; ‘what we're hungry for is good, definitive, evidence-based work that shows… the timeframes of benefits, the savings [that] come through and who they're to’ (int.13). Public health researchers could also develop work with other sectors (e.g. transport) to improve evidence relevant to public health teams' work across LG.^6,37,38^

This study indicates how, during the process of making the case for investment in public health, DsPH are adapting to different organizational norms and values and negotiating perceptions about boundaries of public health practice to show, as Green^39^ suggests, the added value of public health. Challenges around resource allocation will persist. Public health teams require a range of advocacy skills and support (including evidence) to be most effective and thrive in LG; public health training may need to be revisited to reflect the skills required.

## Funding

This work was funded by Public Health England. M.W., W.H. and R.C. work in The Centre for the Development and Evaluation of Complex Interventions for Public Health Improvement (DECIPHer), a UKCRC Public Health Research Centre of Excellence. Joint funding (MR/KO232331/1) from the British Heart Foundation, Cancer Research UK, Economic and Social Research Council, Medical Research Council, the Welsh Government and the Wellcome Trust, under the auspices of the UK Clinical Research Collaboration, is gratefully acknowledged. Views expressed in this paper do not represent those of the funders or the UKCRC. The funders had no role in study design, data collection and analysis, decision to publish or manuscript preparation.

## Authors' contributions

M.W., J.W., W.H. and R.C. drafted the research proposal. M.W. conducted the interviews and led the group discussion with support from J.W. and R.C. M.W. analysed the data. All authors contributed to this paper.

## Conflict of interest

J.W. is currently on secondment to Wiltshire Council from Public Health England. All other authors declare no conflict of interest.
